# The Safety of Selective Use of Splenic Flexure Mobilization in Sigmoid and Rectal Resections—Systematic Review and Meta-Analysis

**DOI:** 10.3390/jcm7110392

**Published:** 2018-10-27

**Authors:** Michał Nowakowski, Piotr Małczak, Magdalena Mizera, Mateusz Rubinkiewicz, Anna Lasek, Mateusz Wierdak, Piotr Major, Andrzej Budzyński, Michał Pędziwiatr

**Affiliations:** 1 Department of Medical Education, Jagiellonian University Medical College, Krakow 31-501, Poland; mmnowakowski@gmail.com; 2 Department of General Surgery, Jagiellonian University Medical College, Krakow 31-501, Poland; pmmalczak@gmail.com (P.M.); magda.mizera.f@gmail.com (M.M.); mrubinkiewicz@gmail.com (M.R.); aniad303@gmail.com (A.L.); wierdakmateusz@poczta.onet.pl (M.W.), majorpiotr@gmail.com (P.M.), andrzej.budzynski@uj.edu.pl (A.B.); 3 Centre for Research, Training and Innovation in Surgery (CERTAIN Surgery), Krakow 31-501, Poland

**Keywords:** meta-analysis, splenic flexure mobilization, colorectal surgery

## Abstract

Background: According to traditional textbooks on surgery, splenic flexure mobilization is suggested as a mandatory part of open rectal resection. However, its use in minimally invasive access seems to be limited. This stage of the procedure is considered difficult in the laparoscopic approach. The aim of this study was to systematically review literature on flexure mobilization and perform meta-analysis. Methods: A systematic review of the literature was performed using the Medline, Embase and Scopus databases to identify all eligible studies that compared patients undergoing rectal or sigmoid resection with or without splenic flexure mobilization. Inclusion criteria: (1) comparison of groups of patients with and without mobilization and (2) reports on overall morbidity, anastomotic leakage, operative time, length of specimen, number of harvested lymph nodes, or length of hospital stay. The outcomes of interest were: operative time, conversion rate, number of lymph nodes harvested, overall morbidity, mortality, leakage rate, reoperation rate, and length of stay. Results: Initial search yielded 2282 studies. In the end, we included 10 studies in the meta-analysis. Splenic flexure is associated with longer operative time (95% confidence interval (CI) 23.61–41.25; *p* < 0.001) and higher rate of anastomotic leakage (risk ratios (RR): 1.02; 95% CI 1.10–3.35; *p* = 0.02), however the length of hospital stay is shorter by 0.42 days. There were no differences in remaining outcomes. Conclusions: Not mobilizing the splenic flexure results in a significantly shorter operative time and a longer length of stay. Further research is required to establish whether flexure mobilization is required in minimally invasive surgery.

## 1. Introduction

According to traditional textbooks on surgery, splenic flexure mobilization (SFM) is suggested as an essential part of open rectal resection [1]. However, an international questionnaire among laparoscopic colorectal surgeons showed that SFM was no longer considered mandatory—only 70% routinely mobilized the splenic flexure [2]. In general, this stage of the procedure is considered difficult in the laparoscopic approach [3]. In common understanding, SFM enables tension-free anastomosis by providing sufficient colonic length and thus decreasing the risk of anastomotic leakage and overall morbidity [4]. In addition, it has been speculated that SFM allows achieving a longer specimen, which is believed to result in more adequate lymphadenectomy, leading to better oncologic outcomes. SFM is responsible for the greater difficulty of the procedure, leading to a prolonged operative time and possibly causes increased blood loss and a higher risk of injury to surrounding organs [5,6]. However, the evidence to support this popular belief is sparse. Therefore, the question arises whether the recommendation to perform SFM routinely during each operation of the rectum is still relevant in the evidence-based surgery era. To answer this question, we have conducted a systematic review of the available literature in order to assess the outcomes of colorectal resection with and without SFM.

## 2. Methods

### 2.1. Study Selection

A systematic review of the literature was performed using the Medline, Embase and Scopus databases to identify all eligible studies that compared patients undergoing rectal or sigmoid resection with or without SFM. The used search terms included: splenic flexure, left flexure, left colon, left hemicolon, mobilisation, and mobilization, freeing and releasing. These terms were combined using Boolean operators “AND” and “OR”. Some references of the acquired articles were also located manually. The most recent search was performed on 26 April 2018. The Ovid search strategy is available in supplementary file 1.

Studies eligible for further analysis had to fulfil the following criteria: (1) comparison of groups of patients with and without SFM and (2) an objective evaluation of overall morbidity, anastomotic leakage, operative time, length of specimen, number of harvested lymph nodes, or length of hospital stay. Full texts and conference abstracts were included. There were no language restrictions. Studies were excluded when there was (1) a lack of comparative data or (2) insufficient data to analyze.

### 2.2. Outcomes of Interest

The outcomes of interest were: operative time, conversion rate, number of lymph nodes harvested, overall morbidity, mortality, leakage rate, reoperation rate, length of stay.

### 2.3. Data Extraction and Quality Assessment

All references were reviewed and evaluated by two teams of two researchers. In cases where there were doubts about eligibility for inclusion, an attempt was made to reach consensus within the group. If no resolution was possible, an arbitrary decision was made by a third reviewer. Data from the included studies were extracted independently by all teams. When available, the following data were extracted: first author, year of publication, country, study design, number of operated subjects, their demographic characteristics, and outcomes of interest.

Studies were evaluated according to the Newcastle–Ottawa Scale (NOS), which consists of three factors: patient selections, comparability of study groups, and assessment of outcomes. A score of 0 to 9 was assigned to each study, and studies achieving a score of 6 or higher were considered high-quality. This study was performed according to the Preferred Reporting Items for Systematic Reviews and Meta-Analyses (PRISMA) guidelines and Meta-Analysis of Observational Studies in Epidemiology (MOOSE) consensus statement [7,8].

### 2.4. Data Analysis

Analysis was performed using RevMan 5.3 (freeware from The Cochrane Collaboration, London, UK). Statistical heterogeneity and inconsistency were measured using Cochran’s Q tests and I^2^, respectively. Qualitative outcomes from individual studies were analyzed to assess individual and pooled risk ratios (RR) with pertinent 95% confidence intervals (CI) favoring patients undergoing revisionary surgery and by means of the Mantel-Haenszel random-effects method. When appropriate, mean and standard deviation were calculated from medians and interquartile ranges using a method proposed by Hozo et al. [9]. Weighted mean differences (WMD) with a 95% CI are presented for quantitative variables using the inverse variance random-effects method. Statistical significance was observed with two-tailed 0.05 level for hypotheses and with 0.10 for heterogeneity testing, while unadjusted *p*-values were reported accordingly. Dichotomous outcome analysis involved subgroup analysis for case-control and case-matched studies.

## 3. Results

**Figure 1:**
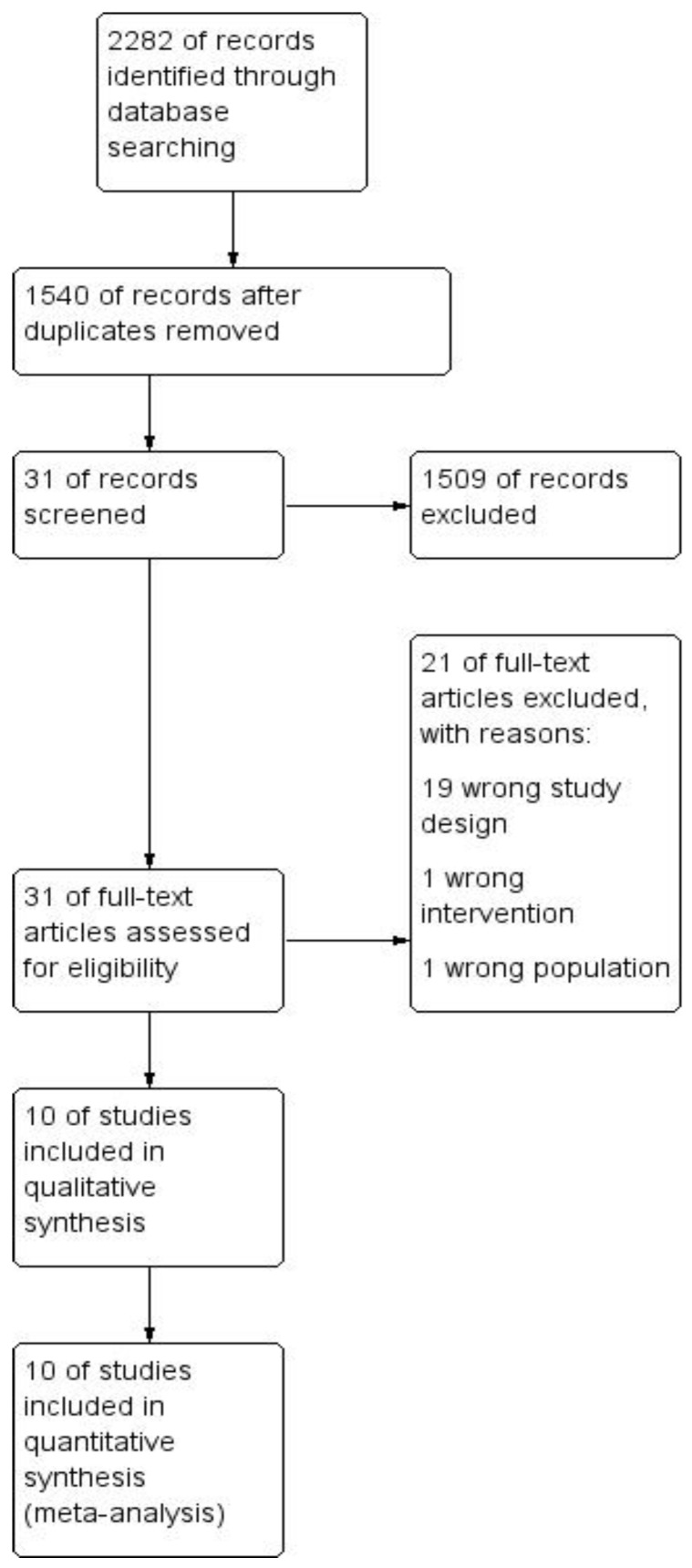
Study selection flow chart.

Our initial search yielded 2282 articles. We recognized 742 duplicates and after removing them we evaluated 1540 articles through titles and abstracts. This produced 31 papers suitable for full-text review. Out of them, there were three conference abstracts with the same data and authors as in the included studies, therefore they were not included in the quantitative analysis. In the end meta-analysis included 10 studies, of which one was a conference abstract not published as a full article. Nine articles were written in English and one in Russian (Figure 1). Our analysis covered 12,944 patients in total (4657 in the SFM (+) group and 8287 patients in the SFM (-) group). A flowchart of the analyzed studies is presented in Figure 1. The majority of studies included cancer patients; the study by Carlson et al. included patients with cancer or non-malignant indications and the study by Schlussel reported non-malignant cases [10,11]. The quality of the included studies according to the NOS scale is low and moderate (highest score 7, lowest 3). Baseline information about the analyzed studies is presented in Table 1.

**Figure 2:**
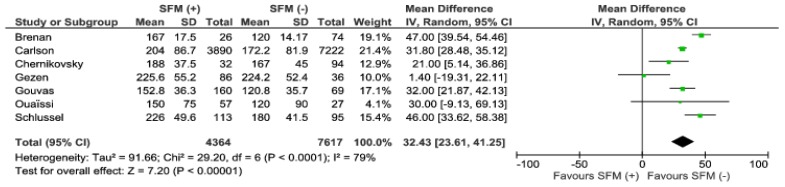
Pooled estimates of operative time comparing SFM (+) versus SFM (−). CI: confidence interval, df: degrees of freedom, SFM (+): splenic flexure mobilization was performed, SFM (−): splenic flexure mobilization was not performed.

Operative time was reported in seven studies. Meta-analysis revealed significant differences in favor for SFM (−) by 32.43 min (95% CI 23.61–41.25; *p* < 0.001), (187.6 min in SFM (+) group vs. 157.7 min in SFM (−)) (Figure 2). Although the heterogeneity of the included studies is high, I^2^ = 79%, only two studies present results which are not in line with the overall results of the meta-analysis [14,18].

**Figure 3:**
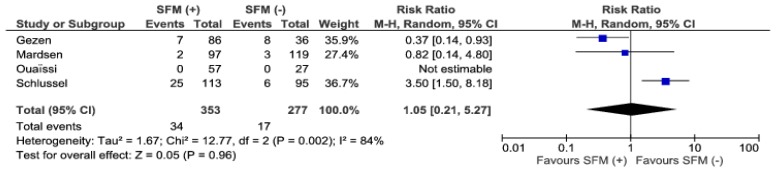
Pooled estimates of conversions comparing SFM (+) versus SFM (−). CI: confidence interval, df: degrees of freedom.

Conversion rates were reported in four studies. We found no significant differences among analyzed groups, 34/353 (9.6%) vs. 17/277 (6.14%), (RR: 1.05; 95% CI 0.21–5.27; *p* = 0.96), however the heterogeneity of the studies is rather high, I^2^ = 84%, (Figure 3).

**Figure 4:**
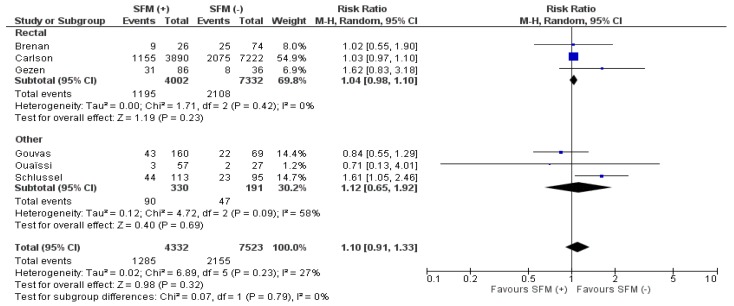
Pooled estimates of overall morbidity comparing SFM (+) versus SFM (−) with subgroup analysis (rectal resections and other resections). CI: confidence interval, df: degrees of freedom.

Overall morbidity was reported in six studies. There were no significant differences between the groups in the analyzed material, 1285/4332 (29.7%) vs. 2155/7523 (28.65%), (RR:1.1; 95% CI 0.91–1.33, *p* = 0.32), (Figure 4). The heterogeneity of the studies is low, I^2^ = 27%. Only one study showed data in favor of the SFM (−) group [11]. Moreover, subgroup analysis of rectal and other resections did not reveal any differences.

**Figure 5:**
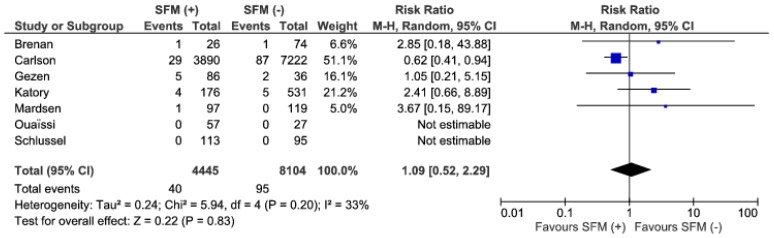
Pooled estimates of mortality comparing SFM (+) versus SFM (−). CI: confidence interval, df: degrees of freedom.

Mortality rates were reported in seven studies. There were no statistically significant differences in the analyzed material, 40/4445 (0.9%) vs. 95/8104 (1.17%), (RR: 1.09; 95% CI 0.52–2.29; *p* = 0.83), (Figure 5). The heterogeneity among the studies is low, I^2^ = 33%. Studies by Ouaissi et al. and Schlussel et al. did not report any mortality in either group. The study by Carslon et al. shows significant differences in favor of SFM (+), 95%CI 0.41–0.94 [10,11,18].

**Figure 6:**
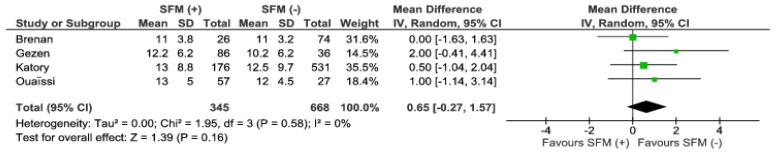
Pooled estimates of lymph node yield comparing SFM (+) versus SFM (−). CI: confidence interval, df: degrees of freedom.

Lymph node yield was reported by four authors. There were no significant differences between analyzed groups, (MD: 0.65; 95% CI −0.28–1.57; *p* = 0.16), (Figure 6). The mean lymph node harvest in the SFM (+) group was 12.6 whereas in the SFM (−) group it was 11.7. Heterogeneity of the studies was low. 

**Figure 7:**
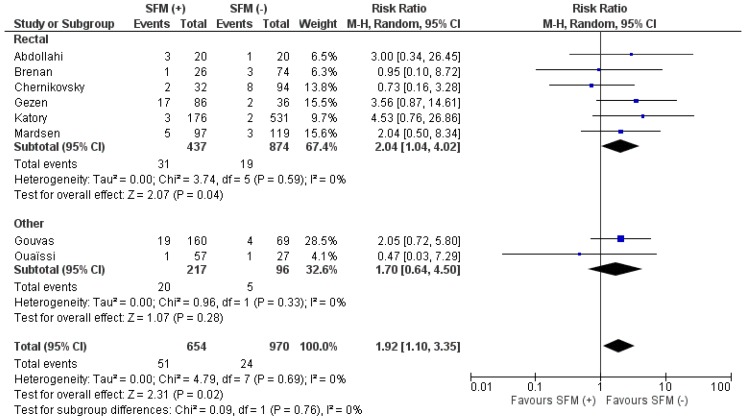
Pooled estimates of anastomotic leakage rate comparing SFM (+) versus SFM (−) with subgroup analysis (rectal resections and other resections). CI: confidence interval, df: degrees of freedom.

Anastomotic leakage was reported in eight studies. Analysis revealed significant differences in favor of SFM (−), 51/654 (7.8%) vs. 24/970 (2.5%), (RR: 1.02; 95% CI 1.10–3.35; *p* = 0.02), (Figure 7). There was no heterogeneity of included studies. These differences were also present when subgroup analysis for rectal resections was performed.

**Figure 8:**
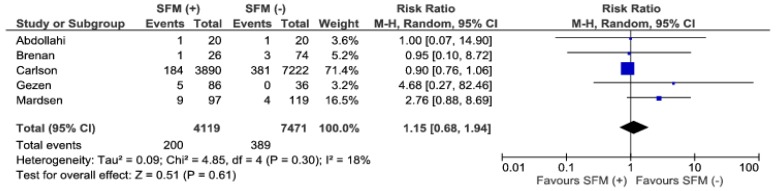
Pooled estimates of reoperations comparing SFM (+) versus SFM (−). CI: confidence interval, df: degrees of freedom.

Reoperations were present in five studies. There were no significant differences among the groups, 200/4119 (4.6%) vs. 389/7471 (5.2%), (RR: 1.15; 95% CI 0.68–1.94; *p* = 0.61), (Figure 8). The heterogeneity of the analyzed material is low, I^2^ = 18%.

**Figure 9:**
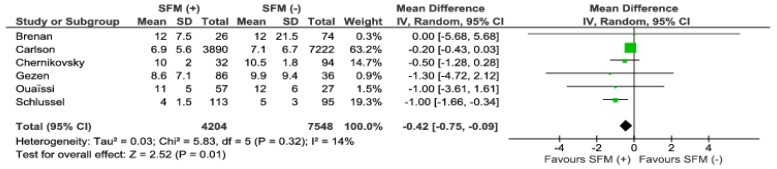
Pooled estimates of length of hospital stay comparing SFM (+) versus SFM (−). CI: confidence interval, df: degrees of freedom.

Length of hospital stay was reported by six authors. Analysis revealed that length of stay (LOS) is shortened by 0.42 days in favor of SFM (+). The mean hospital stay in the SFM (+) group was 8.75 days, whereas in the SFM (−) group it was 9.42 days, (Figure 9). The heterogeneity among the analyzed papers was low, I^2^ = 18%. 

## 4. Discussion

To our knowledge, this is the first systematic review which aims to compare the outcomes of routine versus no splenic flexure mobilization in colorectal surgery. The main findings show that SFM is associated with a longer operative time but also with no differences in the conversion rate, postoperative morbidity and mortality, as well as in lymph node harvest. It is important to note that the leakage rate was higher, and the length of hospital stay was shorter in patients with SFM. However, the moderate and low-quality trials available do not permit for unequivocal conclusions to be drawn.

The ability to create a tension-free anastomosis with an appropriate blood supply and geometry is a matter of the utmost importance in rectosigmoid surgery. Achieving sufficient mobility of bowel endings is a crucial, although not always easy, step in every colorectal operation. According to Jamali, SFM is one of the most demanding parts (after the pelvic part of low anterior resection) of laparoscopic colorectal surgery [3]. As shown in this review, SFM was associated with an operative time which was 32 min longer. Although the heterogeneity of the meta-analysis was high, it was caused by the differences in operative times in the studies under analysis rather than different outcomes. Five out of seven studies showed significant differences in operative time in favor of not mobilizing the splenic flexure. Moreover, there were no differences in conversion rates between groups. This may suggest that SFM, when needed, does not have a clinically relevant impact on operative parameters. Unfortunately, other operative factors (blood loss, intraoperative injury of surrounding organs or other intraoperative adverse events) were reported only in one study and did not show statistical differences; consequently, no reliable conclusions could be drawn.

Postoperative morbidity is the most reliable differentiating parameter in the evaluation of the operative technique. Meta-analysis of the extracted results did not show any differences in the overall morbidity, mortality or reoperation rates. Surprisingly, we noticed that the group without SFM had a lower rate of anastomotic leakage, and this was also present when we performed a subgroup analysis of studies comprising rectal resections. However, we are certain these results should be interpreted with caution. Although (in this aspect) the heterogeneity of the studies under analysis is very low, the total number of patients included is relatively small (654 vs. 970 patients). Calculating the sample size when designing a randomized trial with an anticipated anastomotic leak incidence of 8% and a reduction of 3% as the primary outcome, the minimal study sample should be at least 1059 patients per arm. This shows that there is a risk of a type 1 error in the outcome. Nevertheless, we can conclude that avoidance of SFM is not associated with inferior outcomes, which is an important finding from a clinical point of view. A shorter length of stay after SFM may also be clinically relevant. An interpretation of that finding is difficult due to a large number of factors influencing the LOS. In the studies included in our analysis, the LOS times were 8.75 and 9.42 days, respectively. Surgical centers where modern perioperative clinical pathways were introduced achieved a markedly shorter LOS [20,21,22,23,24]. There are studies showing that SFM may prolong return of bowel movement and prolong LOS [25]. Thus, the exact impact of SFM on this parameter is difficult to establish, as precise information concerning the standards of perioperative care in the studies is missing. It may be speculated that this parameter reflects surgical tradition rather than differences in outcomes.

SFM is believed to facilitate adequate lymphadenectomy, which is supposed to result from high inferior mesenteric artery ligation and dissection along the left colic artery to gain the additional length of colon for tension-free anastomosis [12]. Our meta-analysis demonstrated no differences in this outcome—the number of lymph nodes harvested was similar regardless of SFM. Although this is the only oncologic parameter that was available for extraction in this review, based on the available data, we can assume that SFM has no significant impact on the oncologic effect of resection. Only two studies reported the length of resected specimens, which naturally was longer in cases with SFM but without a documented relationship with long-term survival [12,14]. This obviously requires an in-depth analysis into whether this has an impact on long-term outcomes.

This review has some evident limitations. It comprises only non-randomized studies. As mentioned previously, a well-designed randomized controlled trial would require a relatively large number of patients. Another issue that should be included in planning such a trial (which would be very difficult to perform) is the ratio of patients that would require SFM for technical reasons against randomization. However, information on how many patients, in reality, require SFM to create tension-free anastomosis would be of great benefit. The precise data on this topic are missing; however, our clinical experience tells us that this ratio would be rather high. Another limitation is the lack of sufficient data needed to fully analyze patient characteristic, i.e., their general condition, indications for surgery, location of the tumor, neoadjuvant treatment, defunctioning ileostomy (reported only in one study). All these parameters are well-established factors influencing outcomes and they should be taken into consideration when interpreting results. In all studies, however, the basic characteristics of patients were comparable according to their authors, which to some extent, balances the groups. We included patients undergoing open and laparoscopic surgery. As mentioned previously, SFM seems relatively straightforward in open surgery, and this is not necessarily so in minimally invasive access. Moreover, surgeons’ skills, experience and their personal operative techniques can vary for each approach and for each surgeon. This may also have an important impact on outcomes. The data regarding surgeons’ expertise were, however, not provided in the analyzed studies. On the other hand, recent meta-analyses confirm the equality of minimally invasive access and an open approach in terms of oncologic outcomes [26]. In addition, we included patients undergoing rectal and sigmoid resections either for cancer or benign diseases meaning we did not address low anterior resection exclusively. However, when choosing inclusion criteria and building a search strategy, surgical access and type of surgery were not limited to either of the groups and we decided to follow the initial methodology. Additionally, we did not analyze other factors that might have had an impact on the final results: the surgeon’s experience, high vs. low inferior mesenteric artery ligation, or hospital volume [27].

## 5. Conclusions

This is the first systematic review attempting to assess whether SFM should be mandatory in rectosigmoid surgery. Not mobilizing the splenic flexure results in a significantly shorter operative time and a longer length of stay. There were no differences in conversion rates or overall morbidity and mortality. It also had no impact on the number of lymph nodes harvested. The major limitation of this review is that it consists only of non-randomized trials of moderate quality, comprising, in most cases, a small number of patients. This does not allow firm conclusions to be drawn. Whether reliable randomized trials assessing this topic could be performed is a matter for debate due to the large numbers of patients needed to show the non-inferiority of one technique over another. 

## Figures and Tables

**Table 1:** Baseline study characteristics.

First Author	Year	Country	Study Design	Multi/ Single Centre	Number of Patients SFM (+)	Number of Patients SFM (−)	Female/ Male SFM (+)	Female/ Male SFM (−)	Mean Age SFM +	Mean Age SFM −	Indication Malignant/ Benign SFM (+)	Indication: Malignant/ Benign SFM (−)	(*n*) Type of ProcedureSFM (+)	(*n*) Type of Procedure SFM (−)	LAP/OPEN SFM (+)	LAP/OPEN SFM (−)	Study Quality in NOS Scale
Brennan [12]	2007	Ireland	CS	Single	26	74	10/16	28/46	62.0	54.0	26/0	74/0	16 LAR, 10 AR	42 LAR, 32 AR	0/100	0/100	7
Carlson [10]	2014	USA	CS	Multi	3890	7222	2014/1876	3694/3528	60.0	61.3	1209/2681	2776/4446	3890 LAR	7222 LAR	1939/1951	2849/4373	5
Chernikovsky [13]	2016	Russia	RC	Single	32	94	22/10	56/38	N/A	N/A	32/0	94/0	32 LAR	94 LAR	32/0	94/0	5
Gezen [14]	2012	Turkey	CS	Multi	86	36	29/57	16/20	59.3	55.5	86	36	86 LAR	36 LAR	86/0	36/0	6
Gouvas [15]	2014	Greece	CS	Single	160	69	77/83	36/33	64.5	64.0	160/0	69/0	160 sigmoid	69 sigmoid	160/0	69/0	5
Katory [16]	2008	Singapore	CS	Single	176	531	94/82	259/272	66.0	66.0	176/0	531/0	176 HAR	531 HAR	0/176	0/531	7
Mardsen [17]	2012	UK	CS	Single	97	119	37/60	43/76	N/A	N/A	97/0	119/0	58 LAR, 39 HAR	30 LAR, 89 HAR	44/53	94/25	6
Ouaïssi [18]	2013	France	CS	Single	57	27	36/17	8/19	70.0	69.0	57/0	27/0	57 sigmoid	27 sigmoid	17/36	12/15	7
Schlussel [11]	2017	USA	CS	Multi	113	95	63/50	48/47	57.0	56.0	0/113	0/95	N/A	N/A	88/25	59/36	5
Abdollahi [19]	2014	Iran	Abstract	Single	20	20	N/A	N/A	N/A	N/A	N/A	N/A	20 LAR	20 LAR	N/A	N/A	3

CS: cohort study, RC: retrospective cohort, SFM (+): splenic flexure mobilization was performed, SFM (−): splenic flexure mobilization was not performed, LAP: laparoscopic approach, OPEN: open approach, LAR: low anterior resection with total mesorectal excision, HAR: high anterior resection, AR: anterior resection, sigmoid: sigmoid resection, N/A: not applicable.
